# Radiation Response of Murine Embryonic Stem Cells

**DOI:** 10.3390/cells9071650

**Published:** 2020-07-09

**Authors:** Christine E. Hellweg, Vaibhav Shinde, Sureshkumar Perumal Srinivasan, Margit Henry, Tamara Rotshteyn, Christa Baumstark-Khan, Claudia Schmitz, Sebastian Feles, Luis F. Spitta, Ruth Hemmersbach, Jürgen Hescheler, Agapios Sachinidis

**Affiliations:** 1 German Aerospace Center (DLR), Institute of Aerospace Medicine, Radiation Biology, Linder Höhe, D-51147 Köln, Germany; Christa.Baumstark-Khan@dlr.de (C.B.-K.); Claudia.Schmitz@dlr.de (C.S.); Sebastian.Feles@dlr.de (S.F.); Luis.Spitta@dlr.de (L.F.S.); 2 Curadev Pharma Pvt.Ltd., B87, Sector 83, Noida UP 201305, India; vaibhavsshinde@rediffmail.com; 3 Institute of Neurophysiology and Center for Molecular Medicine Cologne (CMMC), University of Cologne, Robert-Koch-Str. 39, 50931 Cologne, Germany; sureshkp@uni-koeln.de (S.P.S.); akp49@uni-koeln.de (M.H.); trotshte@uni-koeln.de (T.R.); j.hescheler@uni-koeln.de (J.H.); 4 German Aerospace Center (DLR), Institute of Aerospace Medicine, Gravitational Biology, Linder Höhe, D-51147 Köln, Germany; Ruth.Hemmersbach@dlr.de

**Keywords:** embryonic stem cells, radiation, cell viability, cell cycle, gene expression, cell death, Kyoto Encyclopedia of genes and genomes, gene ontology

## Abstract

To understand the mechanisms of disturbed differentiation and development by radiation, murine CGR8 embryonic stem cells (mESCs) were exposed to ionizing radiation and differentiated by forming embryoid bodies (EBs). The colony forming ability test was applied for survival and the MTT test for viability determination after X-irradiation. Cell cycle progression was determined by flow cytometry of propidium iodide-stained cells, and DNA double strand break (DSB) induction and repair by γH2AX immunofluorescence. The radiosensitivity of mESCs was slightly higher compared to the murine osteoblast cell line OCT-1. The viability 72 h after X-irradiation decreased dose-dependently and was higher in the presence of leukemia inhibitory factor (LIF). Cells exposed to 2 or 7 Gy underwent a transient G2 arrest. X-irradiation induced γH2AX foci and they disappeared within 72 h. After 72 h of X-ray exposure, RNA was isolated and analyzed using genome-wide microarrays. The gene expression analysis revealed amongst others a regulation of developmental genes (*Ada, Baz1a, Calcoco2, Htra1, Nefh, S100a6* and *Rassf6*), downregulation of genes involved in glycolysis and pyruvate metabolism whereas upregulation of genes related to the p53 signaling pathway. X-irradiated mESCs formed EBs and differentiated toward cardiomyocytes but their beating frequencies were lower compared to EBs from unirradiated cells. These results suggest that X-irradiation of mESCs deregulate genes related to the developmental process. The most significant biological processes found to be altered by X-irradiation in mESCs were the development of cardiovascular, nervous, circulatory and renal system. These results may explain the X-irradiation induced-embryonic lethality and malformations observed in animal studies.

## 1. Introduction

The genomic integrity of embryonic stem cells is crucial for the fate of embryogenesis. Embryonic stem cells (ESCs) have the potential to differentiate into several cell types and to self-renew [1], allowing the study of developmental processes [1,2,3]. Ionizing radiation is known to interfere with the genomic integrity of ESCs, which may hamper the proper embryogenesis [4] and mutations could also enter the germline. ESCs display a high proliferation rate and a short G1 phase of the cell cycle [5], which might explain their sensitivity to genotoxic stress exerted, e.g., by ionizing radiation [6]. The radiation-induced DNA damage response encompasses cell cycle arrest and DNA repair; failure of these processes can result in cellular senescence or apoptosis. The DNA damage response of ESCs is described to be robust [5]. Nevertheless, during the error-prone process of DNA damage repair, various mutations can arise after exposure to ionizing radiation in different types of cells [7,8].

NASA has already contraindicated human pregnancy in space as various factors like a lack of gravity, exposure to space radiation and adverse climatic conditions might be detrimental to the embryo. The curiosity whether life and reproduction are sustainable under space conditions with the purpose of long-term spaceflight and colonization of other habitable planets and moons in our solar system inspired various aerospace research scientists to investigate the effect of microgravity and radiation on mammalian reproduction. These experiments were performed during space missions as well as in ground-based facilities, using different simulation approaches such as hind limb suspension, clinostats and random positioning machines, thereby missing some lack of comparability [9,10,11,12,13]. Recently it has been proven that the freeze-dried mouse spermatozoa exposed to cosmic radiation on the International Space Station (ISS) for nine months retained the capacity to fertilize female and produce healthy offspring [14]. This indicates a lack of adverse effects of the ISS environment including space radiation on spermatozoa while in the freeze-dried state. The male and female mice or rats sent to space in a biosatellite also revealed fertilization but no births were noted [15]. So, it is important to explore effect of microgravity and radiation on pre- as well as post-implantation embryonic development.

ESCs are pluripotent in nature. Their ability to differentiate in three germ layers makes them an ideal developmental model system to explore potential developmental toxicity mechanisms of (simulated) microgravity and radiation. The main advantage of using murine CGR8 embryonic stem cells (mESCs) is the avoidance of ethical concerns related to human ESCs (hESCs), which have been suggested for risk assessment of ionizing radiation exposure during early human development [16]. The effects of simulated and real microgravity on mESC differentiation [17,18,19] and on human cardiomyocytes derived from human induced pluripotent stem cells (hiPSCs) [20] at the transcriptomics level were reported earlier.

ESCs share some characteristics with cancer stem cells such as the capability for self-renewal [21,22] and are therefore of high interest for cancer research. Cancer stem cells are described to have high tolerance towards DNA damage [5], robust cytoprotective pathways [5] and expression of ESCs factors such as octamer-binding transcription factor 4 (Oct4), which might confer them radioresistance [23]. In contrast, it has been previously shown that mESCs are more sensitive to irradiation with UV light or γ-rays than differentiated mouse embryonic fibroblasts (MEFs) [24], which might be explained by a more open chromatin configuration [24,25] and their high proliferation rate, which goes along with a short G1 phase of the cell cycle [5,26]. In mESCs, a double safeguard mechanism against mutations [24] and unique histone modifications [27] and radio-responses such as reduced ATM activation and ionizing radiation-induced foci [27] were described. On the other hand, the preferential use of homologous recombination (HR) over non-homologous endjoining (NHEJ) by ESCs results in less error-prone repair [28]. For investigating the link between early effects of ionizing radiation exposure such as the initiation of the DNA damage response in ESCs with various outcomes and later alteration of embryonic development, the differentiation of ESCs by the forming of embryoid bodies (EBs) under irradiation can serve as a model and genome-wide gene expression studies (transcriptomics) can contribute to elucidate underlying mechanisms of unbalanced differentiation and toxicity developmental processes.

The aim of this work was to explore the effects of ionizing radiation exposure on the differentiation and developmental processes/signaling pathways of mESCs (CGR8 strain) in an integrated approach covering various aspects of the DNA damages and cell death. To maintain the mESCs in their pluripotent state, they were cultivated in the presence of the leukemia inhibitory factor (LIF). We determined the effect of exposure to various doses of X-rays on cell survival, viability, cell cycle progression, gene expression and the differentiation potential of the mESCs. Viability, cell cycle progression and gene expression were compared for cultivation in the presence and absence of LIF. X-rays were used as reference radiation for future studies with space-relevant radiation qualities such as accelerated heavy ions.

## 2. Materials and Methods

### 2.1. Cell Culture

Murine embryonic stem cells (mESCs; CGR8 strain) were obtained from the European Collection of Cell Cultures (ECACC, Salisbury, UK, No. 95011018). Cells were maintained in the medium for CGR8 (mCM) consisting of Glasgow’s minimum essential medium (Life Technologies, Darmstadt, Germany, No 11710035) with 2 mmol/L glutamine, 50 μmol/L β-mercaptoethanol (β-ME; Life Technologies, Darmstadt, Germany), 1000 units/mL leukemia inhibitory factor (LIF; Merck Chemicals, Darmstadt, Germany), penicillin (100 units/mL)/streptomycin (100 μg/mL) and 10% fetal bovine serum (FBS; GIBCO, Life Technologies, Darmstadt, Germany) at standard conditions (37 °C, 95% air and 5% CO_2_ atmosphere). Cell culture flasks (25 cm^2^, Nunc, Novodirect, Kehl, Germany) were coated with 2 mL of 0.2% gelatin and incubated for 30 min (5% CO_2_, 37 °C). After incubation, the gelatin was aspirated and 5 mL of mCM containing 1 × 10^6^ mESCs was added to each flask. Cells were passaged at the sub-confluent stage (70–80%). The medium was exchanged after 2–3 days.

### 2.2. Treatment Modalities

For X-irradiation experiments, 1 × 10^4^ cells/cm^2^ were seeded into suitable culture vessels (Petri dishes ∅ 3 cm or 6 cm, Nunc; Costar 9102 96-strip well plates, Sigma-Aldrich Chemie, Steinheim, Germany; 25 cm^2^ flasks, Nunc) freshly coated with gelatin (0.2%, Sigma-Aldrich Chemie) for 30 min at 37 °C, and grown for two days before treatment.

X-irradiation: cells in the exponential growth phase were exposed at room temperature (RT) to low linear energy transfer (LET; 0.3–3.0 keV/µm) X-rays using the X-ray source RS225 (Gulmay Medical, now: X-Strahl, Surrey, UK) at DLR Cologne. The X-ray tube was adjusted at a voltage of 200 kV and a current of 15 mA. Soft X-rays were eliminated by using a copper (Cu) filter of 0.5 mm thickness. Dose and dose rate were determined using the dosimeter UNIDOS^webline^ with the ionizing chamber TM30013 (PTW, Freiburg, Germany). The distance of the samples from the X-ray source was set at 420 mm to provide a constant dose rate of 1.0 Gy/min. After irradiation, samples were transferred to the incubator and harvested at different time intervals according to the experimental requirements.

### 2.3. Colony Forming Ability

To determine survival after X-rays exposure, cells were irradiated in 12.5 cm^2^ flasks as confluent cell layers as described in 2.2. X-irradiation. After irradiation, cells were trypsinized and seeded into Petri dishes (∅ 6 cm, Nunc, Roskilde, Denmark). The cell number in the dishes was adjusted to compensate for the plating efficiency of the cell line and for the anticipated lethal effect of the radiation in order to allow growth of about 50 colonies per dish.

The cells were incubated for 12 days without medium change. The resulting colonies were stained with crystal violet (1 mg/mL in 3.5% formaldehyde solution) for 45 min. After microscopic control, only colonies containing more than 50 cells were scored as survivors. The experiments were performed with six dishes per dose and repeated independently three times.

All data from the irradiated samples were fit by a least-squares linear regression analysis to ln(N/N_0_) versus dose and analyzed following the single hit multi-target model.

### 2.4. MTT Test

The mESCs were seeded into gelatin-coated 96-strip well plates at a density of 1000 cells per well (3000 cells/cm^2^) in 200 µL of mCM medium per well. 24 h later, cells were irradiated with X-rays (see 2.2) or exposed to 10% ethanol as a positive control for cytotoxic effects. After 72 h, cells in one strip were killed by adding pure dimethyl sulfoxide (DMSO, Sigma-Aldrich Chemie) to generate the blank, medium was exchanged in all wells by serum free medium containing 0.1 mg/mL MTT reagent (3-(4,5-dimethylthiazole-2-yl)-2,5-diphenyl tetrazolium bromide, M5655, Sigma-Aldrich Chemie) and cells were incubated at 37 °C for 1 h. The medium was removed and the formazan crystals were dissolved in DMSO containing 0.6% *v/v* acetic acid and 10% *w/v* SDS (sodium dodecyl sulphate, Sigma-Aldrich, St. Louis, MO, USA) by mixing for 20 min. Absorption was measured at 562 nm in a microplate reader (Lambda Fluoro 320 plus, MWG Biotech, Ebersberg, Germany). Viability was calculated according to the following formula (1):

(1) $$Viability\left(\%\right)=\frac{OD_{treated}-OD_{blank}}{OD_{untreated}-OD_{blank}}\times 100$$

OD = optical density at 562 nm

### 2.5. Cell Cycle Analysis

For cell cycle analysis, cells were seeded into petri dishes (∅ 3 cm, Nunc) in the presence or absence of LIF at a density of 1 × 10^4^ cells/cm^2^ and grown for one day. After X-irradiation, the medium supernatant was collected and cells were washed with phosphate-buffered saline (PBS) and detached with trypsin-EDTA (Pan-Biotech, Aidenbach, Germany). Cells in the medium supernatant and in the washing solution and trypsinized cells (total volume 1.5 mL) were fixed by addition of 4.5 mL ice-cold ethanol (100%, stored at −20 °C) at specific time points and stored at −20 °C. Fixed samples were prepared for flow cytometry by adding two volumes of PBS (1:3 dilution of ethanol). Cells were pelleted by centrifugation (500× *g*, 5 min). The cell pellet was suspended in DNA staining solution (50 μg/mL RNAse A, VWR Chemicals, Darmstadt, Germany; 0.1% Triton X-100, Sigma-Aldrich Chemie; 20 μg/mL propidium iodide, Calbiochem part of Merck, Darmstadt, Germany; in PBS) for 1 h at 37 °C. The propidium iodide fluorescence was measured in a flow cytometer (FACScan, Becton Dickinson, San Jose, CA, USA) in the FL-2 channel. For cell doublet discrimination, width and area of the FL-2 signal were recorded. The data were analyzed by Flowing Software 2.5.1 (Perttu Terho, Turku Centre for Biotechnology, Finland, https://bioscience.fi/services/cell-imaging/flowing-software/) and the cell cycle analysis Microsoft Excel sheet developed by Prof. Dr. Christa Baumstark-Khan (described in [18]).

### 2.6. Immunofluorescence Staining of γH2AX

The mESCs were seeded at a density of 3 × 10^4^ cells/cm^2^ on gelatin coated cover slips and cultivated in Petri dishes (∅ 3 cm, 2 mL 0.2% gelatin per Petri dish) in the presence or absence of LIF. Cells were X-irradiated (0, 1, 2 and 7 Gy) 24 h later as described in 2.2 and incubated until fixation after 0.5, 24 or 72 h. Cells were washed with 0.5 mL Tris-buffered saline with 0.05% Tween-20 (TBS-T) and fixed with 0.5 mL TBS + 0.05% Tween-20, 3.5% formaldehyde and 3 mmol/L magnesium chloride for 10 min at 4 °C. The fixation solution was removed and cells were washed with TBS-T and stored at 4 °C until immunofluorescence staining.

The cover slips were washed three times with TBS-T. Cells were permeabilized with 0.5% Triton X-100 in PBS containing 1% bovine serum albumin (BSA) for 20 min on ice. After three washing steps in TBS-T, cells were incubated with the primary antibody (anti-γ-H2AX polyclonal rabbit serum 1:150, R&D Systems, Minneapolis, MN, USA, No AF2288) for 45 min at room temperature and 99% humidity. The antibodies were diluted in Antibody Diluent (Zytomed Systems, Berlin, Germany). Unspecific binding was blocked simultaneously during the incubation through the Antibody Diluent. The cover slips were washed three times with TBS-T, the last time for 10 min. The incubation with the secondary antibody (NL557 donkey-anti-rabbit antibody, 1:300, R&D Systems, No NL004) lasted 45 min at room temperature, 99% humidity and protected from light. After thorough washing, cell nuclei were stained with 0.1 µg/mL DAPI (4′,6-Diamidino-2-phenylindole dihydrochloride, Sigma-Aldrich Chemie) for 15 min at room temperature. The cover slips were mounted on slides using the fluorescent mounting medium (Dako, Santa Clara, CA, USA).

Images of the blue and red fluorescence were taken at constant illumination times using the fluorescence microscope M1 Imager (Zeiss, Oberkochen, Germany) with the camera AxioCam MRm (Zeiss, Oberkochen, Germany) using the filter sets filter set 43 HE (Ex BP 550/25, Em BP 605/70, DsRed, Zeiss, Oberkochen, Germany) and filter set 49 (Ex G 365, Em BP 445/50, DAPI, Zeiss, Oberkochen, Germany).

### 2.7. Differentiation of mESCs

Differentiation of mESCs was performed and embryoid bodies (EBs) were formed by a hanging drop culture. Drops from a 20 mL suspension containing 400 cells were placed on the inner surface of the lid of a sterile square plate (12 cm × 12 cm × 1.7 cm; Greiner Bio-One, Kremsmünster, Austria, No 688102). The square plate contained 5 mL PBS. Differentiation started by the formation of EBs after the lids were slowly inverted and kept on the square plate for different periods. Then all square plates were gently placed inside the CO_2_ incubator. The EBs were collected on day 3 and transferred to sterile petri plates (10 cm diameter, Greiner Bio-One) containing random differentiation medium (Iscove’s Modified Dulbecco’s Medium, IMDM, supplemented with 20% FBS, 100 mmol/L β-ME, 2 mmol/L L-glutamine and 1% non-essential amino acids (NEAA) (vol/vol), Life Technologies). The plates containing EBs were placed on a continuously shaking shaker inside a CO_2_ incubator (reciprocation motion 50/min). The medium was changed every second day. On day 10, videos of beating EBs were captured and samples were collected for further analysis.

### 2.8. RNA Isolation and Microarray Preparation

RNA isolation from the samples was performed as previously reported [18]. Briefly, total RNA was isolated using TRIzol and chloroform (Sigma-Aldrich Chemie) and purified with the miRNeasy mini kit (Qiagen, Hilden, Germany). The quantification was done using a NanoDrop (ND-1000, Thermo-Fisher, Langenselbold, Germany). For microarray labeling 100 ng of the total RNA was taken as a starting material and after amplification 12.5 µg of amplified RNA was hybridized on Mouse Genome 430 version 2.0 arrays (Affymetrix, Santa Clara, CA, USA) for 16 h at 45 °C. The arrays were washed and stained in Affymetrix Fluidics Station-450 according to the manufacturer’s instructions. After staining, arrays were scanned with Affymetrix Gene-Chip Scanner-3000-7G and Affymetrix GCOS software was used for quality control analysis.

### 2.9. Gene Expression Analysis

For gene expression analysis the .CEL files obtained from the microarray experiments were uploaded in Partek Genomics Suite (PGS) version 6.6 (Partek, St Louis, MO, USA). The probe sets (PS) intensity values were generated by robust multi-array average (RMA) background correction, quantile normalization, log2 transformation and median polished probe sets (PS) summarization. The normalized PS was used for the generation of principal component analysis (PCA). The one-way ANOVA model was used to generate the differentially regulated transcripts with at least a 2 fold change (*p* ≤ 0.05). The signals of differentially regulated transcripts were normalized by the Z score and clustered using a hierarchal cluster analysis (unsupervised, PGS). The online free software Database for Annotation, Visualization and Integrated Discovery (DAVID) was used for functional annotation and gene ontology categories (GOs) of differentially expressed genes. Furthermore, the files were imported into CellNet, a network biology platform [29], to diagnose differences in the gene regulatory networks that are active in the mock-irradiated and X-irradiated mESCs and in the absence or presence of LIF [30,31].

### 2.10. Statistics

Each experiment was repeated up to five times with one to six replicates each. In order to account for different numbers of replicates and repeats, the standard error was calculated. Means, standard errors and significance levels in the *t* test were calculated with Microsoft^®^ Office Excel 2003. Regression analyses and 95% confidential belts were performed using SigmaPlot 13.0.

## 3. Results

In order to characterize the cellular responses of mESCs to X-rays exposure, survival, viability, cell cycle progression, gene expression and induction of γH2AX foci as an indicator of the DNA double strand breaks were analyzed. Effects of X-irradiation on the differentiation potential of mESCs were assessed by generating EBs using the hanging drop differentiation protocol as described previously [18] toward cardiomyocytes and other somatic cells.

### 3.1. Survival and Viability of mESCs after X-rays Exposure

**Figure 1:**
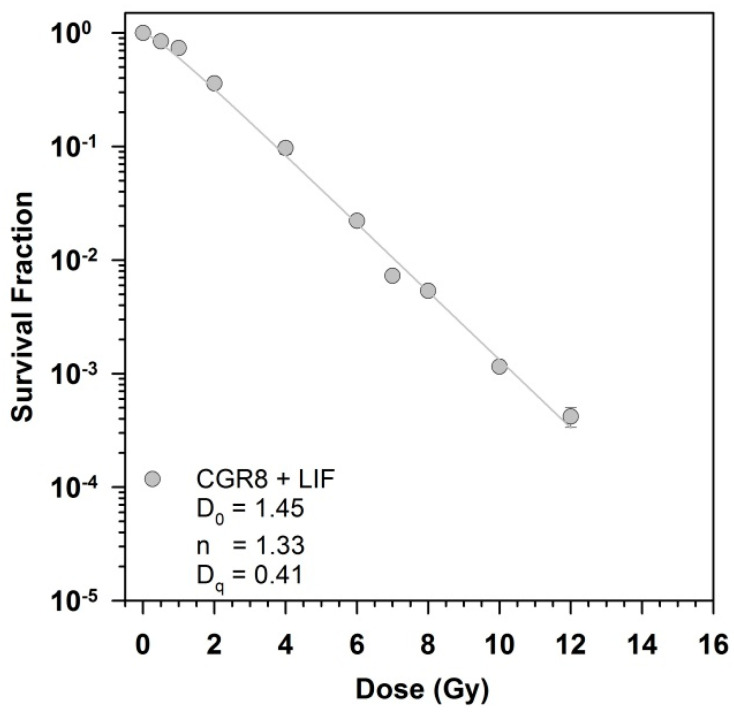
Clonogenic survival after exposure of murine CGR8 embryonic stem cells (mESCs) to X-rays. Survival was determined by the colony forming ability test (CFA). The cells were seeded in flasks, irradiated after reaching confluence, plated in Petri dishes immediately after irradiation and colonies were fixed and stained after 11 days incubation in presence of the leukemia inhibitory factor (LIF). The parameters D_0_ (the reciprocal of the slope in the linear range of the survival curves) and the extrapolation number n of the dose effect curve were calculated by a regression analysis (survival versus dose). The quasi threshold dose Dq was derived from the intersection of the extrapolated linear part of the survival curve with the 100% survival line. The survival data were fitted to the equation $S=1-{\left(1-e^{-\frac{D}{D0}}\right)}^{n}$. The data of three independent experiments with 6 replicates were combined; bars show the standard error, if they are not visible, they are smaller than the symbol.

Exposure to X-rays in the presence of LIF resulted in a shouldered survival curve indicating cellular DNA damage repair capacity (Figure 1). The plating efficiency of mESCs in gelatin-coated petri dishes was 0.64 ± 0.11.

**Figure 2:**
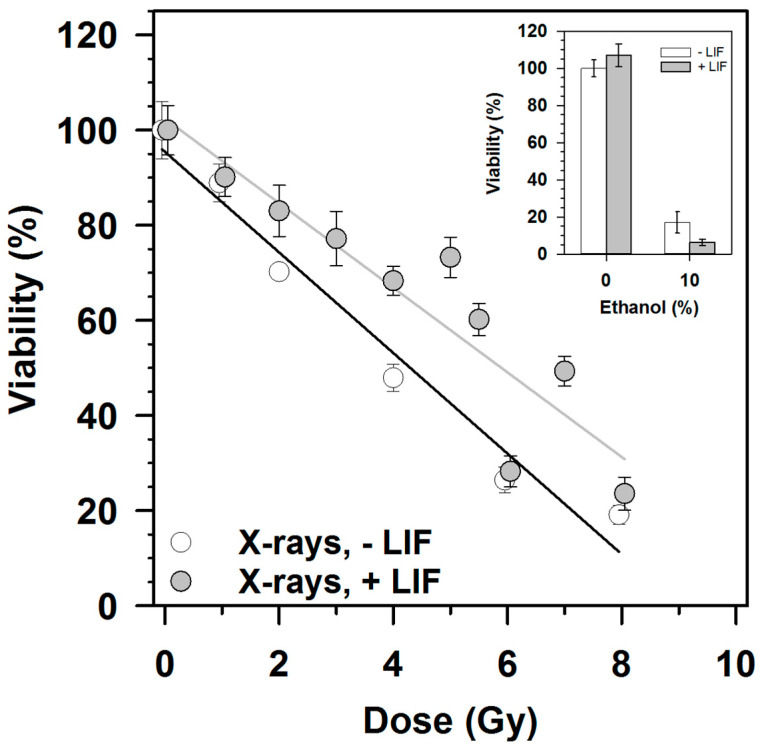
Viability after exposure of mESCs to 200 kV X-rays. Viability was determined by the MTT test. The cells were seeded, irradiated 24 h later and the MTT test was performed after 3 days incubation. Data of three independent experiments were combined; bars show the standard error of 12 wells (4 per independent experiment). The small inset shows the reduction of viability after cell incubation with 10% ethanol, as positive control for a cytotoxic effect, in the absence or presence of LIF. *p* < 0.05 (*t*-test) for 4 Gy, - LIF/+ LIF.

The viability decreased dose-dependently up to 8 Gy three days after X-irradiation, as detected with the MTT test (Figure 2). The decrease was stronger when cells were incubated without LIF compared to growth in the presence of LIF. This difference reached significance at a dose of 4 Gy.

Doses higher than 8 Gy did not result in further reduction of viability below 20%, as the limit of quantification of the MTT test was reached in this viability range. Treatment with 10% ethanol for 72 h reduced the viability strongly.

### 3.2. Cell Cycle Progression after X-ray Exposure

**Figure 3:**
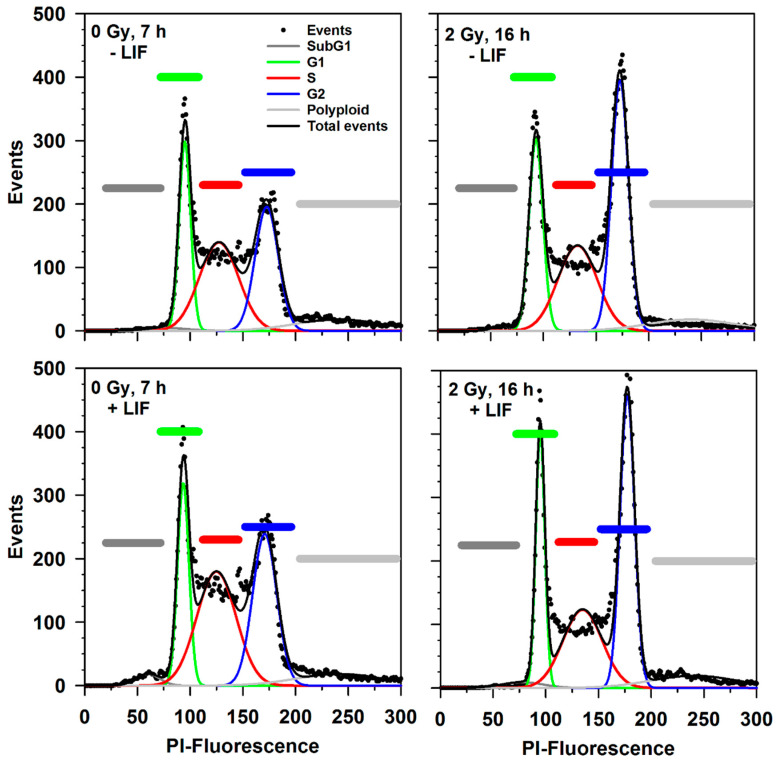
Determination of cell cycle progression after X-ray exposure (0 Gy and 2 Gy). The identification of the subpopulations with sub-G1, G1, S and G2 phase or polyploid DNA content is shown in exemplary propidium iodide (PI) fluorescence histograms. mESCs were incubated in the absence (-LIF) or presence of LIF (+ LIF) for 7 h (left) or 16 h (right). Cellular DNA was stained with propidium iodide, RNA was digested by incubation with RNase. Propidium iodide fluorescence was determined by flow cytometry.

**Figure 4:**
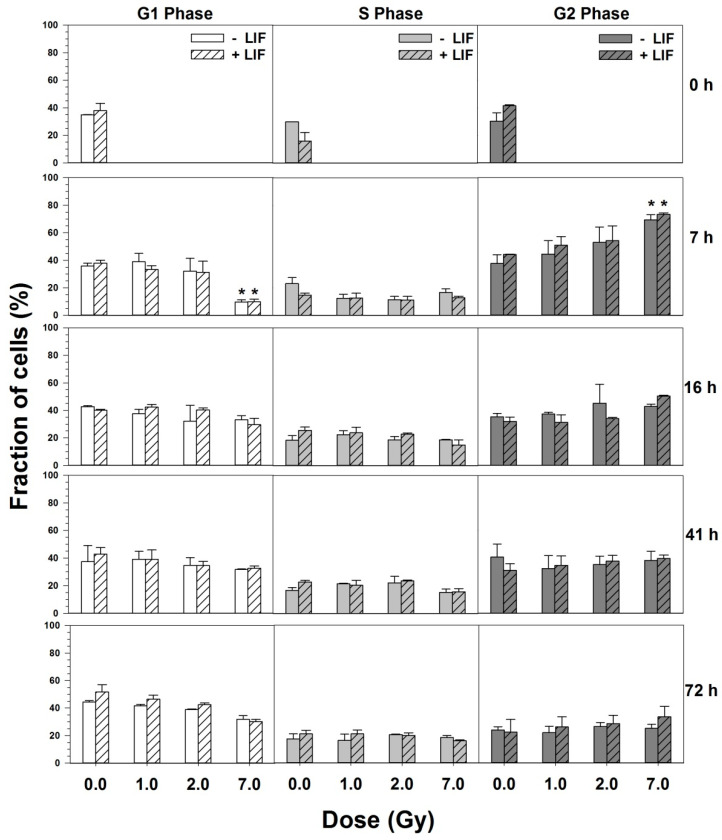
Cell cycle progression of mESCs after X-ray exposure. Cells were X-irradiated one day after seeding. At the indicated time-points, cells were trypsinized and fixed in 70% ethanol. Cellular DNA was stained with propidium iodide; RNA was digested by incubation with RNase. Propidium iodide fluorescence was determined by flow cytometry and cells were assigned to the different cell cycle phases based on their DNA content. * *p* < 0.05 (*t*-test) for irradiated vs. mock-irradiated.

Cell cycle progression after X-irradiation was followed by flow cytometry of ethanol-fixed and propidium iodide-stained cells (Figure 3, Figure 4). Figure 3 shows four propidium iodide fluorescence histograms containing data from single cells. The percentage of cells in the different cell cycle phases (G1, S and G2), of cells with a sub-G1 DNA content (apoptotic cells) and of polyploid cells was determined by step-wise identification of the cell populations implying the PI histogram to represent the sum of the five respective Gaussian distributions for the subpopulations. This was achieved by using the generalized reduced gradient (GRG) non-linear algorithm with the Solver function of a Microsoft Excel Macro Sheet-based analysis program. Based on an user-defined estimated value for the G1 peak position and peak maximum starting values for determining the positions and peak maxima of the other subpopulations were iteratively determined and the Gaussian distributions were calculated until the minimum for the least squares of the errors for the sum of the five distributions was found to be minimized.

24 h after seeding, 33–35% of the cells were in the G1 phase, 22–28% in the S-phase and 33–38% in the G2 phase (Figure 4). The cell cycle distribution did not differ significantly in the presence or absence of LIF. After X-irradiation, a dose-dependent transient arrest in the G2 phase occurred as indicated by the accumulation of cells in the G2 phase. The dose-dependent increase of cells in the G2-phase 7 h and 12 h after irradiation was accompanied by a decrease of cells in the G1 and S phases.

These changes reached significance at a dose of 7 Gy. The G2 arrest was diminished after 16 h, and further reduced after 41 h, and not significant anymore. Three days after irradiation, 31–47% of the cells were in the G1 phase, 17–24% in the S-phase and 24–33% in the G2 phase. No significant differences in cell cycle progression were observed in the absence or presence of LIF.

**Figure 5:**
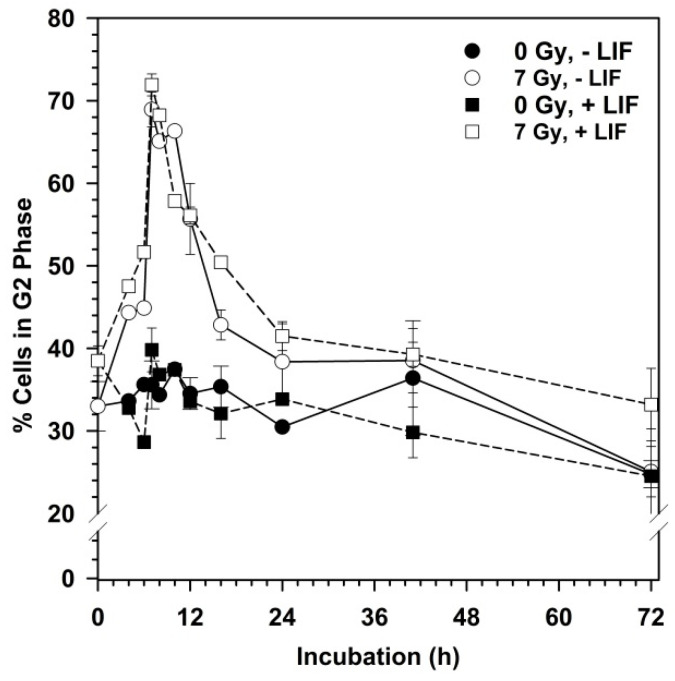
Kinetics of G2 arrest after exposure of mESCs to 7 Gy X-rays. Cells were irradiated one day after seeding and cultivating with or without LIF for up to 72 h. Cells were trypsinized and fixed in 70% ethanol at the indicated time-points. Cellular DNA was stained with propidium iodide; RNA was digested by incubation with RNase. Propidium iodide fluorescence was determined by flow cytometry and cells were assigned to the different cell cycle phases based on their DNA content.

In Figure 5, the kinetics of the G2 arrest after exposure of mESCs to 7 Gy X-rays is displayed. This demonstrated the sharp increase in the number of cells in the G2 phase within the first 7 h after irradiation, and a fast resolution of the G2 arrest within the next 17 h. The G2 arrest was completely resolved after 41 h.

### 3.3. γH2AX as Marker of the Cellular Response to DNA Double Strand Breaks

**Figure 6:**
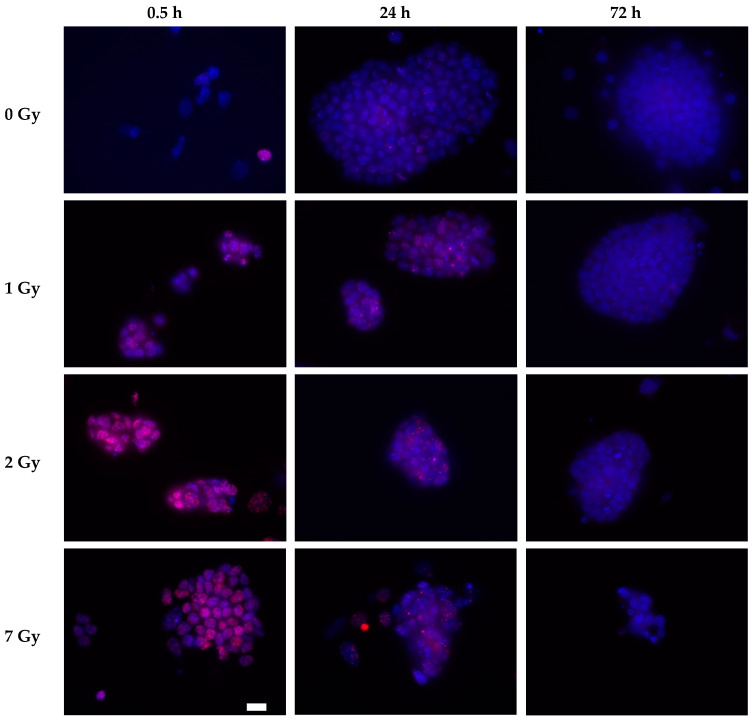
γH2AX foci in mESCs after exposure to X-rays in the presence of LIF. Cells were seeded on cover slips, grown for one day and exposed to X-rays. Cells were fixed with 3.5% formaldehyde at the indicated time points. Immunofluorescence staining was performed using a γH2AX antibody (pink foci), and cell nuclei were stained with DAPI (blue). Scale bar (white box) 20 µm.

**Figure 7:**
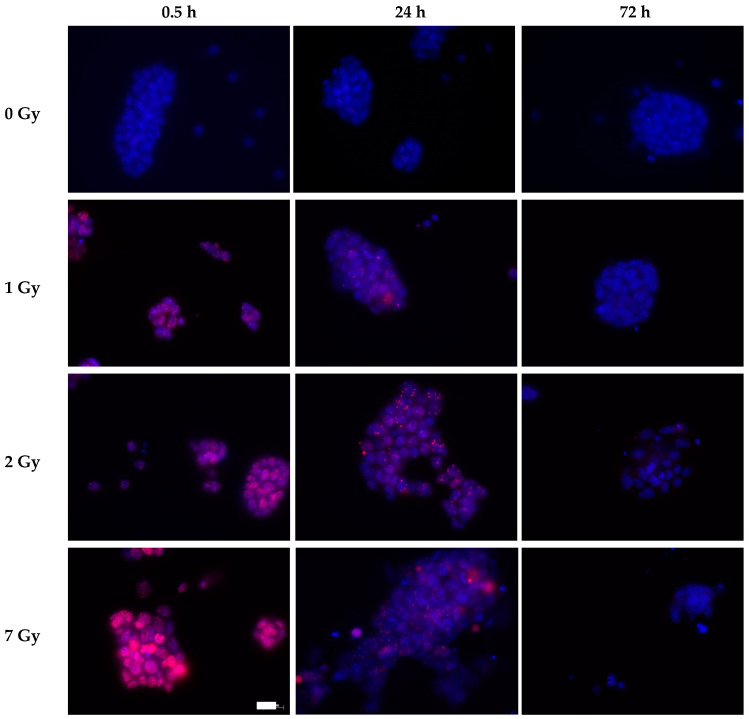
Representative images of γH2AX foci in mESCs after exposure to X-rays in the absence of LIF. Cells were seeded on cover slips, grown for one day and exposed to X-rays. Cells were fixed with 3.5% formaldehyde at the indicated time points. Immunofluorescence staining was performed using a γH2AX antibody (pink foci), and cell nuclei were stained with DAPI (blue). Scale bar (white box) 20 µm.

The S139 phosphorylated form of the histone variant H2AX, γH2AX, was detected in the cell nuclei of irradiated cells 30 min after irradiation (Figure 6, Figure 7). The fluorescence intensity increased with increasing dose. In the control cells (0 Gy), only a few cells displayed γH2AX foci. In irradiated cells, residual γH2AX fluorescence was detected 24 h after irradiation. These foci resolved within the next 2 days, resulting in nearly foci-free cell nuclei 72 h after irradiation. No obvious difference in γH2AX fluorescence was observed for cultivation with and without LIF (Figure 6, Figure 7).

### 3.4. Data Structure of X-ray Deregulated Genes in mESCs Cultured in Presence and Absence of LIF

**Figure 8:**
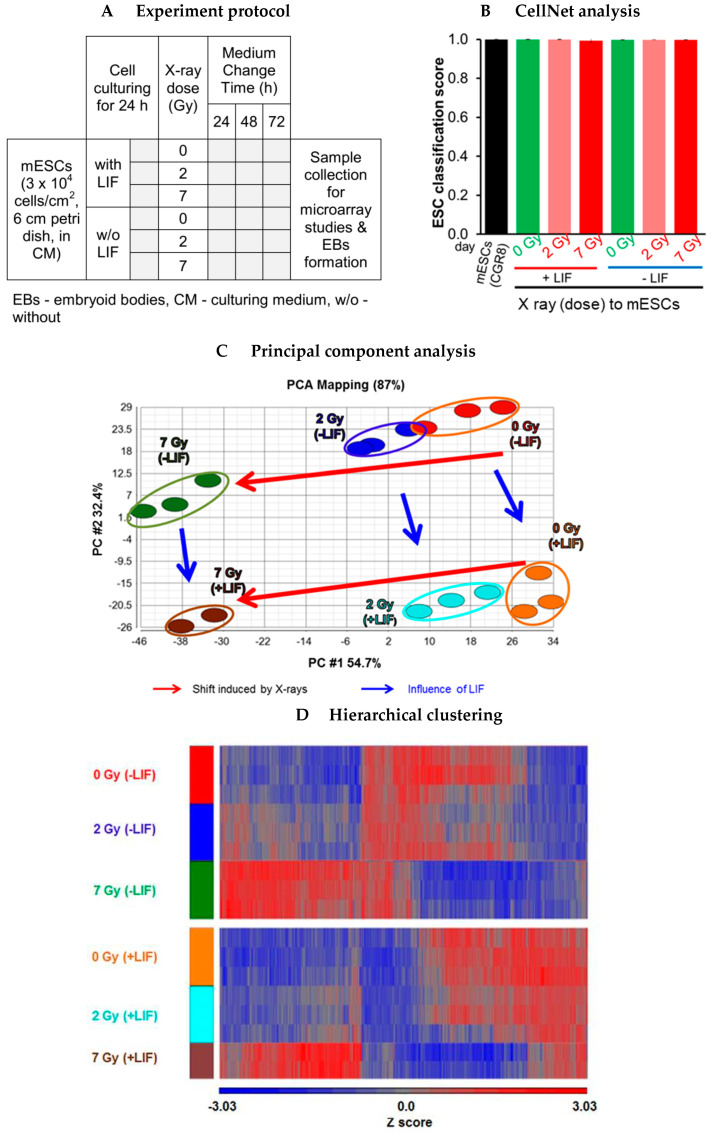
Gene expression of mESCs after X-rays exposure. (**A**) mESCs cells were cultured on Petri dishes for 24 h and then exposed to X-rays doses of 0 Gy, 2 Gy and 7 Gy. The cells were then maintained in the presence or absence of leukemia inhibitory factor (LIF) for 72 h. The cells were collected after 72 h for microarray studies and embryoid bodies (EBs) formation. (**B**) CellNet analysis revealed a uniform ESC classification score for mESCs exposed to 0 Gy, 2 Gy and 7 Gy and further cultured for 72 h in the presence and absence of LIF. (**C**) The data structure of transcriptome (data sets based on X-ray deregulated genes (fold change ≥ ±2, *p* < 0.05) was dimensionality-reduced and presented in form of a 2D principle component analysis (PCA) diagram. The PCA illustrates a distance along PC #1, which primarily denotes an X-rays-induced shift whereas PC #2 represents the LIF-induced shift. (**D**) All transcripts whose expression was significantly modified (fold change ≥ ±2, *p* < 0.05) after exposure to X-rays were used for the hierarchical cluster analysis (unsupervised, Partek Genomics Suite (PGS)). The results are represented as a heat map with each row representing one experiment, each column indicating data for one probe set and the color of each cell indicating the row-wise z-score of gene expression levels (blue: decreased low; red: increased). The data represent the mean of three independent experiments.

mESCs were cultured in the presence of LIF for 24 h, exposed to 0 Gy, 2 Gy and 7 Gy and further cultured for 72 h in the presence and absence of LIF (Figure 8A). The microscopic photographs and samples were collected at 72 h. RNA was isolated from the samples for the analysis of gene expression using microarrays.

#### 3.4.1. Microscopic Observations

**Figure 9:**
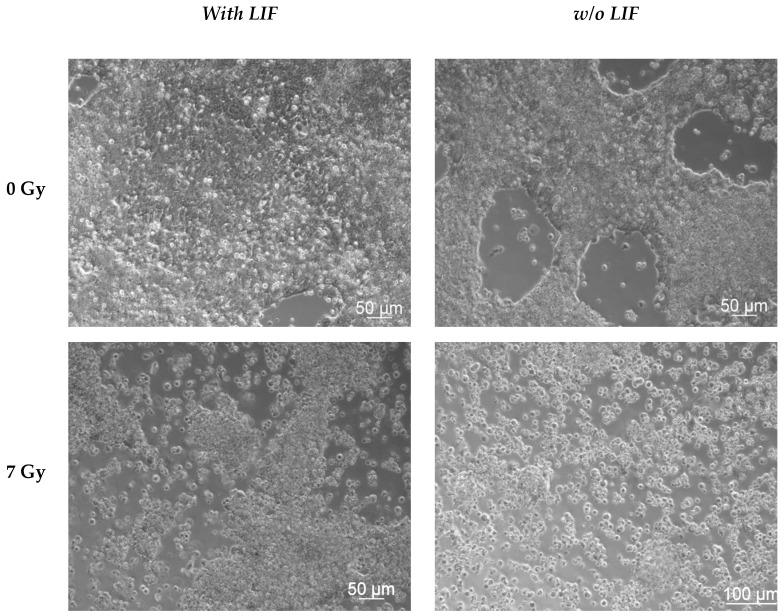
Morphology of mESCs after exposure to X-rays. mESCs cells were cultured in Petri dishes for 24 h and then exposed to X-rays (0 Gy and 7 Gy). The cells were then maintained in the presence and absence of the leukemia inhibitory factor (LIF) for 72 h. The cells were visualized 72 h after irradiation by phase contrast microscopy.

The microscopic observations (Figure 9) revealed a nearly confluent CGR8 cell layer after mock-irradiation (0 Gy) and incubation with LIF. After 72 h incubation without LIF, the cell layer was subconfluent. After exposure to 7 Gy X-rays, surviving cells could not produce a confluent cell layer within 72 h, irrespective of LIF presence or absence. This indicates that the cell proliferation was proficiently hampered by the X-ray exposure.

#### 3.4.2. CellNet Analysis

To compare the influence of X-rays on pluripotency and to quantify resemblance with mESCs, we performed a CellNet analysis of the so-called .CEL files obtained from a microarray study [30,32]. CellNet provides a platform for quantifying how closely engineered cell populations resemble their target cell type. For the ESC classification score, it compares the gene regulatory network (GRN) of uploaded files with expression data compiled from 56 published study data sets and provides the ESC score as output. An ESC classification score > 0.98 was reached for all samples (Figure 8B) indicating that the pluripotency network in mESCs was very similar to 56 published data sets of ESCs, independent of the treatment. This result indicates that the gene regulatory network related to pluripotency was not much influenced by X-rays or by LIF 72 h after exposure.

#### 3.4.3. Overview of Differentially Regulated Genes

The overview of deregulated genes between various groups was observed by the principle component analysis (PCA) plot (Figure 8C). The principle component 1 (PC #1) along the x-axis represents the X-rays-induced shift, whereas PC #2 represents the shift induced by the presence and absence of LIF. Irrespective of the presence of LIF, the relative distance between cells exposed to 0 Gy and 7 Gy, respectively, was larger than that of cells irradiated with 0 Gy and 2 Gy, indicating a dose-dependent effect of X-rays on gene expression. Overall the shift induced by X-rays was stronger than the LIF induced shift.

#### 3.4.4. Developmental Genes and X-ray Influenced Genes

The list of differentially regulated genes between the groups is presented in Supplementary Table S1 (having an absolute fold change ≥ 2 and False Discovery Rate (FDR)-corrected *p*-value < 0.05). Hereafter, the genes differentially regulated in the absence of LIF (0 Gy) with respect to the presence of LIF (0 Gy) will be designated as developmental genes whereas the genes differentially regulated between X-rays exposed cells (7 Gy and 2 Gy) with respect to the control (0 Gy) will be called X-ray influenced genes. The hierarchical clustering of developmental genes and X-ray influenced genes based on the Z score is presented in Figure 8D. The mESCs exposed to 7 Gy X-rays were clustered together irrespective of presence of LIF indicating a high number of deregulated genes. The other two groups (0 Gy and 2 Gy) were clustered together based on the presence or absence of LIF, which indicated less impact of 2 Gy on gene deregulation.

#### 3.4.5. Overlap Analysis of Developmental Genes with X-ray Influenced Genes and Biological Processes Influenced (GOs)

**Figure 10:**
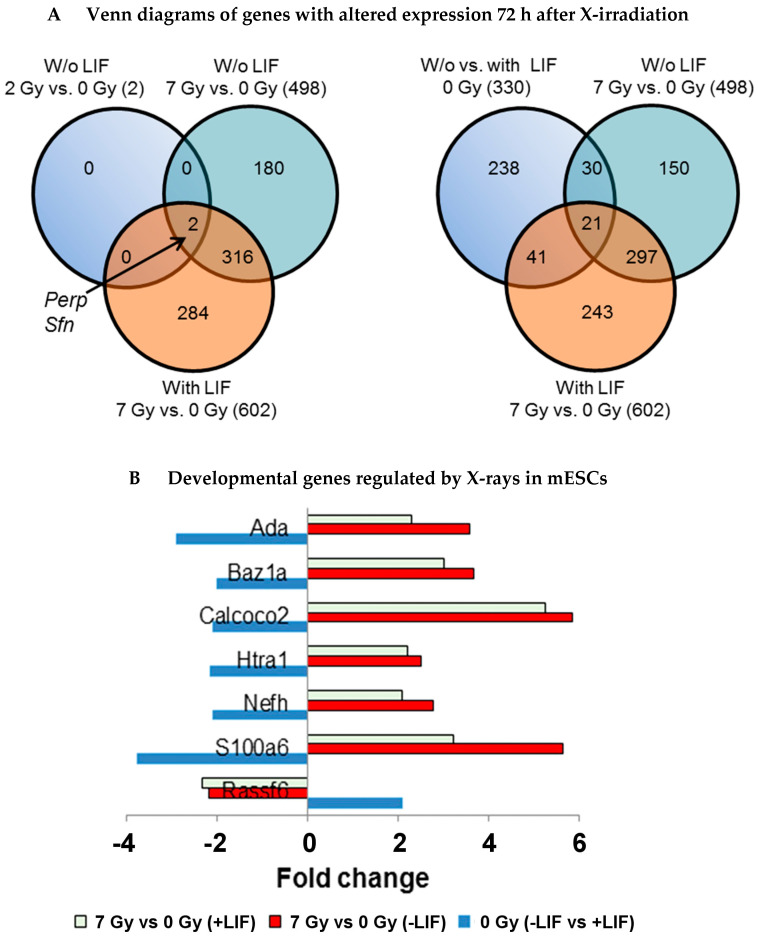
Characterization of X-ray deregulated genes. (**A**) The overlap analysis of the total number of genes whose expression was altered after exposure to X-rays and differentially expressed genes in the absence or presence of LIF with respect to 0 Gy (fold change ≥ ± 2, False Discovery Rate (FDR) corrected *p*-value < 0.05, detailed data are shown in supplemental material). (**B**) Developmental genes (0 Gy, *w/o* LIF vs. LIF) regulated by X-rays in the presence or absence of LIF.

The overlap analysis of X-ray influenced genes (Figure 10, Supplementary Table S1) indicated that two common genes Perp and Sfn (14-3-3 σ) were influenced by 2 Gy (without LIF) and 7 Gy doses (with and without LIF). The mESCs cultured in the presence of LIF after exposure to 2 Gy X-rays did not significantly deregulate any genes. Overlap analysis of X-rays influenced genes (7 Gy) with the developmental genes indicated the common 21 developmental genes influenced by 7 Gy exposure (Figure 10). The 297 common genes were deregulated by 7 Gy in the presence and absence of LIF whereas 243 genes were deregulated specifically in the presence of LIF and 150 genes in the absence of LIF (Figure 10). This indicates that after X-irradiation, the fate of gene regulation greatly depended on the presence or absence of LIF.

Seven genes from the common 21 developmental genes were regulated by X-irradiation (Figure 10B). These genes are namely *Ada, Baz1a, Calcoco2, Htra1, Nefh, S100a6* and *Rassf6.*

#### 3.4.6. Overlap Analysis of Biological Process Gene Ontologies (GOs) Influenced by Deregulated Genes

**Figure 11:**
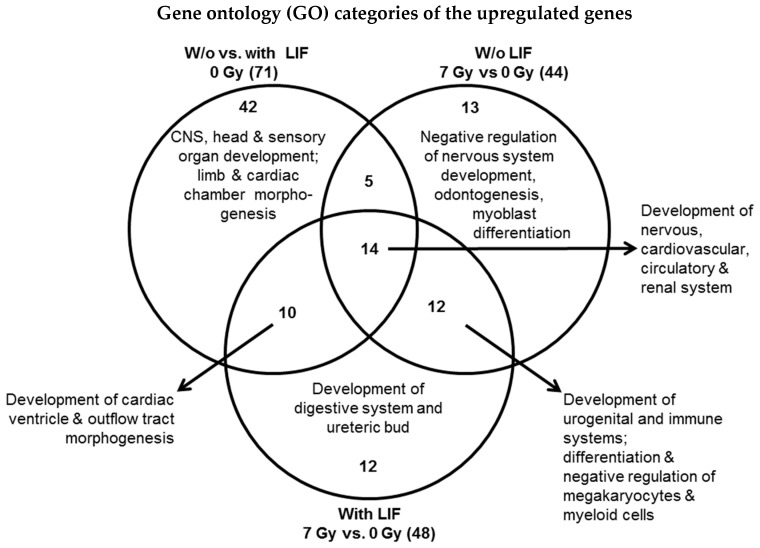
Venn diagram of biological processes altered by developmental genes and X-ray deregulated genes. The overlap analysis of gene ontology (GO) categories belonging to specific embryonic development shows the overrepresented categories amongst upregulated genes (fold change ≥ ±2, *p* < 0.05, detailed data are shown in supplemental material).

For characterization of the genes with expression that was significantly modified by X-irradiation, the biological processes including development in which these genes are involved were captured by uploading the gene list in the online data analysis software “DAVID” (Supplementary Table S2). LIF mostly affected genes that are involved in the embryonic development. Exposure to X-rays also upregulated genes of the embryonic development and it downregulated genes majorly involved in metabolic processes related to carboxylic acid, organic acid, nicotinamide nucleotide, lipid, etc. The biological gene ontology (GO) processes (GOs, by upregulated genes) were further filtered based on the specific embryonic development and the overlap analysis was performed (Figure 11). The common embryonic development processes related to the development of nervous, cardiovascular, circulatory and renal systems were modified by LIF as well as X-rays exposure. X-ray exposed mESCs cultured in the presence of LIF additionally revealed upregulated genes belonging to the digestive system and the ureteric bud development related GOs. The upregulated genes related to negative regulation of nervous system development, odontogenesis and myoblast differentiation was observed in mESCs exposed to X-rays and further cultured in the absence of LIF (Figure 11).

#### 3.4.7. The Kyoto Encyclopedia of Genes and Genomes (KEGG) Pathways and Chromosome Location of Developmental Genes and X-ray Influenced Genes

The p53 signaling pathway was activated by X-rays in both conditions (presence/absence of LIF) whereas the absence of LIF further influenced the Hippo signaling and the PI3K-Akt signaling pathways. The genes downregulated X-rays belonged to pathways related to “glycolysis, pyruvate metabolism and biosynthesis of amino acids” (Table 1, Supplementary Table S3).

**Figure 12:**
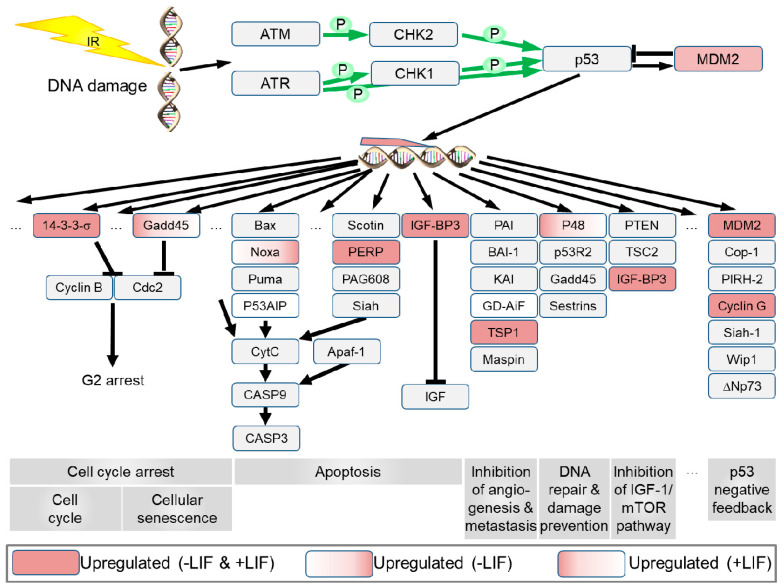
Influence of X-rays (7 Gy) on regulation of the p53 pathway (according to the *Mus musculus* p53 signaling pathway available at https://www.genome.jp/kegg/) [33] in mESCs in presence of LIF (+LIF) and absence of LIF (-LIF). Upregulated genes are highlighted in red. IR, ionizing radiation; …, genes not shown; genes in white boxes are not yet established in mice.

X-rays upregulated more than >30 genes, which resided on chromosome 3 of mESCs (± LIF), whereas 42 genes resided on chromosome no. 13 of mESCs (+ LIF, Table 2). According to the KEGG pathway published by Kanehisa Laboratories (https://www.genome.jp/kegg/) [33], the X-ray-induced genes predominantly captured in the p53 signaling pathway in the presence or absence of LIF with few differences of gene expression observed in both conditions (Figure 12). The genes growth arrest and DNA-damage-inducible, Gamma (*Gadd45g*) and DNA damage-binding protein 2 (*Ddb2*, protein P48) were upregulated in the presence of LIF, whereas phorbol-12-myristate-13-acetate-induced protein 1 (*Pmaip1* or *Noxa*) was upregulated in the absence of LIF (Figure 12).

### 3.5. Fate of X-ray Exposed mESCs after Differentiation towards Beating Cardiomyocytes

EBs were formed from X-ray exposed mESCs and were cultured for 10 days. There was no significant difference observed for beating of EBs on day 10 of differentiation, although there was a visual difference observed between the EBs formed after 7 Gy exposed mESCs and cultured with LIF for 72 h with respect to 0 Gy. The enhanced beating was observed in those EBs (Supplementary Video 1 WA3_0Gy_Exp1 and Supplementary Video 2 WC3_7Gy_Exp1).

## 4. Discussion

### 4.1. Clonogenic Survival and Viability

Based on a D_0_ of 1.45 Gy, the radiosensitivity of mESCs is in the range of the D_0_ normal mammalian cells, which spreads from 1 to 2 Gy. In contrast, the D_0_ of radiosensitive cells amounts to 0.5 Gy. Compared to a non-stem cell line, the murine calvaria-derived osteoblast-like cell line OCT-1, the D_0_ of mESCs was lower (1.76 Gy OCT-1 vs. 1.45 Gy CGR8) [34]. The quasi-threshold dose Dq of mESCs was much lower compared to OCT-1 cells (0.41 vs. 2.78 Gy) [34]. This low Dq indicates the narrow shoulder of the mESCs X-ray survival curve. A survival curve of mESCs that displays a very small shoulder is in line with earlier results [35,36].

As a D_0_ is not available in other studies of mESCs clonogenic survival after ionizing radiation exposure, the survival at an ionizing radiation dose of 4 Gy was compared. It was 0.1 for the mESCs used in this study and for mESCs of the R1 strain analyzed by others, whereby the plating efficiency of the R1 cell line was lower (0.40 ± 0.11) compared to mESCs (0.64 ± 0.11) [37]. A comparable survival level after exposure to 4 Gy was also found in the mESC line LK-1 [38] and for other mESCs strains [39,40] while it was a little lower in the mESC line EDJ22 (0.05) [27] and in IB10 ESCs [41]. For CCE mESCs, a dose of 5.4 Gy γ-rays was required to reduce the survival to 10% [42]. In wild-type TC1 mESCs, around 60% of the cells succumbed to reproductive cell death after exposure to 4 Gy γ-rays, resulting in a survival of 40% [43]. In a C57bl/6 mice wild-type ESCs, the survival after exposure to 4 Gy γ-rays was around 70% [44] and therefore even higher compared to TC1 mESCs. Around 70% survival was also observed after exposure of mESCs to 3.8 Gy γ-rays [45]. In summary, the survival after exposure to 4 Gy ionizing radiation ranges from 5 to 70% for different mESC lines.

The viability of mESCs 72 h after exposure to 1 Gy X-rays was above 90%, independent of the presence of LIF. For comparison, the viability of H9 human (h)ESCs 65 h after exposure to 1 Gy γ-rays amounted to 83%, as determined by live staining with Hoechst 33342 and propidium iodide [46]. In this work, a dose-dependent decrease in viability in the dose range of 1–8 Gy was observed, with no further decrease at higher doses as the limit of quantification of the MTT assay was reached. In contrast, in the colony forming ability test, the survival fraction decreased further after exposures to doses > 8 Gy, which is due to the fact that the survival assay reflects the unlimited cell division capability of the cells, as for the formation of colonies at least five cell divisions are required. The MTT assay 72 h after irradiation detected the number of viable cells at this time point. This cell number is determined by the short-term release of cells from a radiation-induced cell cycle block and the number of cells surviving the radiation exposure. The presence of LIF slightly increased the viability after X-irradiation (Figure 2), which might be explained by the fast proliferation in the presence of LIF while removal of LIF initiates differentiation [47].

### 4.2. Cell Cycle Progression and DNA Repair

A transient G2 arrest after exposure to higher X-ray doses and the lack of a G1 arrest were observed in mESCs after X-irradiation. In other studies, exposure of mouse ESCs to 4 Gy or 2 Gy γ-irradiation resulted in a transient G2 arrest, which resolved within 24–96 h, while a small fraction of cells remained in the G2 block 96 h after exposure to 5 Gy γ-rays [48,49]. A C57bl/6 wild-type ESC line responded to 5 Gy γ-irradiation initially (6 h) by accumulation in the S phase and G2 leading to a depletion of cells in the G1 pool, followed by a strong increase in cells in the G2 fraction (at 12 h) and in the case of γ-rays normalization of the G1 pool at 24 h [44].

hESCs temporarily arrested the cell cycle in the G2 stage following 2 Gy of γ-radiation, and also not in the G1 stage [50,51]. This G2 arrest resolved after 16 h and the arrest was ATM-dependent [51]. In the hESC line H1, a transient G2 arrest with a peak at 2 h after exposure to 5 Gy γ-rays was reported [52]. In hESCs H9 cells, exposure to 1 Gy γ-rays resulted in a G2 arrest 4 h after exposure, which was not completely resolved 24 h after irradiation [53]. This observation was in line with a transient upregulation of CDKN1A and GADD45A [53].

A temporary G2 arrest of ESCs via ATM was described by Stambrook and Tichy [54,55]. Some studies with mESCs revealed a lack of ATM activation after exposure to ionizing radiation [34,52,53], while others report ATM foci formation in mESC after ionizing radiation exposure [6]. A low phosphorylation of p53 and strong phosphorylation of ATM was reported in mESCs after γ-irradiation (1 Gy) [6]. In contrast, according to Jacobs et al. [25], the ATM activation is reduced in stem cells.

Earlier experiments with BRCA1 deficient ESCs have shown that BRCA1 is involved in the maintenance of ionizing radiation-induced G2/M arrest via p53 dependent upregulation of the 14-3-3σ (Sfn) gene [56]. In these ESCs, around 80% of the cell population was accumulated in the G2/M phase of the cell cycle 12 h after γ-irradiation (10 Gy), and the percentage decreased slowly after reaching this peak [56]. The role of *rad1* in the maintenance of a γ-ray-induced (10 Gy) G2 block was shown using *rad1*-deficient (*Mrad^−/−^*) mESCs [57].

Embryonic stem cells do not arrest in the G1 phase [6,52,56], which might be due to a nonfunctional p53-p21 pathway at the G1/S checkpoint [50,58] or high abundance of the Cdc25A phosphatase [59] or a lack of Cdc25A-cyclin-dependent kinase 2 (Cdk2) communication [60] by sequestration of Chk2 kinase at centrosomes and its unavailability for phosphorylation of Cdc25A phosphatase [61]. Additionally, a low expression of the p53 target gene p21/Waf1 was found in mESCs after γ-irradiation [5]. In addition, ESCs lack a p53-dependent intra-S phase arrest [5,59].

The PI3-like protein kinases ATM/ATR and DNA-dependent protein kinase catalytic subunit (DNA-PKcs) catalyze S139 phosphorylation of H2AX (γH2AX) [62]. In the immunofluorescence staining, γH2AX foci indicate sites of DNA DSB [63,64]. In mESCs, X-irradiation resulted in an increase of fluorescence intensity indicating phosphorylation of H2AX to γH2AX and the fluorescence intensity was reduced over time (Figure 6, Figure 7). Additionally, in response to γ-irradiation, γH2AX foci formed in mESCs [6]. The residual damage after 24 h might indicate a delay in DNA repair, which is resolved after 72 h (Figure 6, Figure 7). However, in a study comparing DNA double strand break (DSB) repair in murine embryonic fibroblasts (MEFs) and ESCs based on γH2AX foci counting, no significant difference in the number of foci was found 1–12 h after exposure to 4 Gy γ-rays, indicating comparable repair capacities [48]. In contrast, in R1 mESCs, repair of DNA DSBs was only 60% complete 24 h after exposure to an extremely high X-ray dose (75 Gy), as determined by the neutral comet assay, while in MEFs, only 10% residual DNA DSBs were observed [37]. Removal of LIF inducing differentiation of mESCs improved DNA DSBs repair [37]. Compared to the highest dose of this work (7 Gy), the lowest dose of the dose-effect curve, 10 Gy, did not reveal a defect in the repair of X-ray-induced DNA DSBs [37]. In hESCs, the foci formation and repair capacity is again described differently. A higher level of DSBs, based on detection of γH2AX 1 h after X-irradiation, was found in hESCs compared to human induced pluripotent stem cells (hiPSCs) and primary human dermal fibroblasts [50]. The DNA damage was repaired by hESCs (BG01, I6) with enhanced capacities compared to differentiated cells [65]. mESCs and hESCs are suggested to repair DSB preferably via homologous recombination [37,66,67].

Additionally, in the mock-irradiated mESCs, cells with a multitude of γH2AX foci were found (Figure 6). A higher basal level of these foci was reported for LF2 mESCs in comparison to endothelial cells and smooth muscle cells that were differentiated from the LF2 mESCs and this was explained by a higher replicative stress in mESCs resulting in replication-associated DNA DSBs compared to differentiated cells [68]. This mitotic stress can be very high in some embryonic regions during early embryogenesis—divisions can occur every 2–3 h [44]. Disturbed DNA repair and cell cycle arrest may result in the loss of cells during early embryogenesis when the total cell number is still small. Therefore, the G2 arrest and potentially error-free repair via homologous recombination support ESCs in maintaining their genomic integrity.

### 4.3. Differentiation and Gene Expression Changes

Radiation-induced embryonic lethality and malformations were reported with in vivo mouse studies. The most radiosensitive period was 1–7 h post-fertilization. X-ray exposure during this period was embryonic lethal, whereas exposure to 1.5 day old embryos resulted in either mortality or malformations (exencephaly, polydactyly, eye defects, cleft palate, etc.) in surviving fetuses. The early lethality was attributed to inability of a one cell embryo to recover from G2 arrest after irradiation [69]. So, the reported malformations occurred in the embryos that recovered from the G2 cell cycle arrest and probably due to altered spatial–temporal expression of genes during development. In the present study, we also observed that mESCs were temporarily arrested in the G2 phase after X-irradiation and they completely recovered from the arrest 72 h after exposure. We obtained the transcriptomic profile of these cells at 72 h after exposure, which helped us to understand which gene sets are deregulated by X-irradiation and may contribute to the embryonic malformations.

The CellNet analysis revealed a similar ESC classification score (>0.98) for X-ray-exposed and un-exposed cells 72 h after exposure for both, the absence and presence of LIF. In the X-ray-exposed cells, the DNA strand breaks were either repaired or the cells were sent to apoptosis during first 41 h after exposure, so the cells obtained at 72 h might have conserved their genomic integrity and at the same time X-irradiation had not significantly altered the expression of pluripotent genes. This data is consistent with the studies concluding unaltered expression of pluripotency genes in mESCs and hESCs after X- or γ-irradiation [70,71].

LIF is considered to be important for maintenance of the pluripotent state of mESC, its removal for 21 h from the culture medium resulted in downregulation of pluripotency genes like Klf4 and Tbx3 [47]. In the present study, these genes were also found to be downregulated after LIF removal for 3 days. In consistency with earlier studies reported [70,71], we also found that X-irradiation did not alter the expression of these pluripotency genes in mESCs. Additionally, exposure of murine blastocysts to 0.1 Gy, 0.5 Gy or 1 Gy X-rays did not alter the expression of *Oct4*, *Cdx2* and *Nanog* [72]. In hESCs, exposure to up to 4 Gy did not change the expression of pluripotency genes [73], or the change was only transient, as reported for a decrease in *Oct4*-mRNA after exposure to 2 Gy [51].

In the present study, we observed that X-irradiation regulated seven developmental genes (*Ada, Baz1a, Calcoco2, Htra1, Nefh, S100a6* and *Rassf6*) in mESCs. We state that although microarray data revealed deregulation of developmental genes, some caution for conclusions on the relative expression levels is required as data need further to be validated by qPCR for this. In earlier studies, we validated the expression of some other genes that was quantified with the Mouse Genome 430 version 2.0 arrays with qPCR and the expression pattern was similar for both methods [18].

A literature survey revealed the importance of these genes in the embryonic development and in disease pathology. The adenosine deaminase gene (*Ada*) plays a protective role in early post-implantation embryonic development [74]. Expression of the *Htra1* (Htra serine peptidase 1) gene is spatial–temporally controlled in mouse embryonic development; low expression levels were observed in early organogenesis whereas increased levels were observed in late organogenesis in various tissues [75]. In the present study, withdrawal of LIF from mESCs resulted in downregulation of *Htra1* expression, whereas X-irradiation resulted in upregulation of its levels. Gene *Baz1a* (Bromodomain adjacent to zinc finger domain 1 A) plays a role in the expression of genes important for the nervous system development in mouse embryos [76]. Mutations in the *NEFH* (neurofilament, heavy polypeptide) gene interfere with neurofilament assembly and cause neurotoxicity resulting in disorders of peripheral nervous system in humans [77]. *Nefh* homozygous knockout mice exhibit decreased axon diameters. *Nefh* over-expression in transgenic mice resulted in the development of a neurological defect [78]. Altered expression of *CALCOCO2* was reported in cardiomyopathy [79].

Even though X-irradiation (7 Gy) did not alter the expression of pluripotency genes, it altered the expression of >400 other genes involved in development or homeostasis (498 without LIF, 602 with LIF). These genes were mapped to the biological processes they belong. It was observed that the common biological process captured by these genes was the development of the nervous system, the cardiovascular system, circulatory system and the renal system. In vivo studies with in utero X-irradiation of mice fetuses at different days of gestation resulted in malformations like stunted growth, deformed head, exencephaly, cardiac hernia, skeletal malformation, etc. [80]. These malformations were different with the time of gestation for X-irradiation. The X-irradiation of mouse embryos in utero at the onset of neurogenesis resulted in apoptosis of differentiating neurons and disruption of mouse brain development [81]. Our study was planned to study the effect of X-rays on mESCs and its further differentiation. A further time kinetic study with X-ray exposure during differentiation of mESCs will help to link altered gene expression with specific types of malformation that were observed.

Seven or eight genes of the p53 signaling pathway were still upregulated 72 h after exposure of mESCs to 7 Gy X-rays, in the presence or absence of LIF, respectively. A strong activation of p53 dependent, predominantly pro-apoptotic genes was observed in hESCs H9 cells 2 h after exposure to 1 Gy γ-radiation, which disappeared from the list of over-represented biological pathways already 16 h after exposure [53]. In mESC LF2, an increase of S15 phosphorylated p53 was reported to peak 1 h after exposure to 10 Gy ionizing radiation, and the level decreased gradually over the next 5 h [68]. Nuclear accumulation of p53 in mESCs reached a peak 4 h after exposure to 7.5 Gy γ-rays and reappeared in the cell nuclei 24 h after irradiation [82]. This biphasic p53 activation might be responsible for the upregulation of p53-dependent genes 72 h after X-irradiation that was observed in this work. In response to DNA damage, p53 activation might activate differentiation-associated genes [83].

## 5. Conclusions

X- irradiation of mESCs resulted in DNA double strand breaks, cell cycle arrest in the G2 phase for 16 h after irradiation and cell mortality in a dose-dependent manner and those were independent of LIF’s presence or absence. After successful DNA repair these cells were completely released from the G2 block at 72 h. X-irradiation had no effect on the expression levels of pluripotency markers. X-irradiation altered >400 genes, which were related to various biological processes like the development of nervous, cardiovascular, circulatory and renal systems. Of particular interest were seven developmental genes, which were negatively regulated by X-ray irradiation. Out of these *Htra1, Baz1a, Ada, Nefh* and *Calcoco2* genes were reported to be involved in vivo mouse embryonic development. *Ada* plays a protective role in the embryo (post implantation), *Baz1a* and *Nefh* are related to nervous system development whereas *Calcoco2* is related to cardiac system development. In vivo studies revealed radiation induced embryonic lethality and malformations (exencephaly cleft palate, polydactyly, eye defects, etc.) in mice. Our results indicate that X-irradiation of mESCs deregulated genes related to these developmental process, X-irradiation of specific lineages during differentiation of mESCs will further shed light on radiation induced embryonic lethality and malformations in mice.

## Figures and Tables

**Table 1:** Main KEGG pathways influenced by X-irradiation * of mESCs (± LIF).

Group	KEGG Pathway (Up)	Total Genes	*p* Value	KEGG Pathway (Down)	Total Genes	*p* Value
w/o LIF 7 Gy vs. 0 Gy	p53 signaling	7	7.74 × 10^−4^	Glycolysis	8	3.62 × 10^−6^
	Hippo signaling	11	1.80 × 10^−4^	Pyruvate metabolism	6	3.82 × 10^−5^
	PI3K-Akt signaling	15	1.81 × 10^−3^	Biosynthesis of amino acids	7	1.17 × 10^−4^
with LIF 7 Gy vs. 0 Gy	p53 signaling	8	2.13 × 10^−4^	Glycolysis	8	4.31 × 10^−5^
				Pyruvate metabolism	5	2.34 × 10^−3^
				Biosynthesis of amino acids	8	1.27 × 10^−4^
w/o LIF 0 Gy vs. with LIF 0 Gy	Axon guidance	5	1.97 × 10^−2^	Pluripotency signaling	7	3.17 × 10^−3^
				Osteoclast differentiation	6	1.02 × 10^−2^

* 72 h after irradiation.

**Table 2:** Chromosome location of genes with expression that was modified by X-irradiation * or/and LIF in mESCs.

Group	Gene Regulation	Total Genes	Chromosome Number
w/o LIF 7 Gy vs. 0 Gy	Up	32	3
with LIF 7 Gy vs. 0 Gy	Up	43	3
	Up	42	13
w/o LIF 0 Gy vs. with LIF 0 Gy	Up	13	15
	Down	21	11

* 72 h after irradiation.

## Supplementary Materials

The following materials are available online at https://www.mdpi.com/2073-4409/9/7/1650/s1, Supplementary Table S1: Probe sets (PSs) significantly deregulated at 72 h after exposure of mESCs to X-rays, Supplementary Table S2: The significant biological processes GOs captured by deregulated genes at 72 h after exposure of mESCs to X-rays, Supplementary Table S3: The significant KEGG pathways captured by deregulated genes at 72 h after exposure of mESCs to X-rays, Supplementary Table S4: The significant chromosomes captured by deregulated genes at 72 h after exposure of mESCs to X-rays, Supplementary Video 1: Beating EBs obtained from X-irradiated mESCs WA3_0Gy_Exp1.avi (with LIF 0 Gy), Supplementary Video 2: Beating EBs obtained from X-irradiated mESCs WC3_7Gy_Exp1.avi (With LIF 7 Gy).
